# Cyclic fractionation process for *Saccharina latissima* using aqueous chelator and ion exchange resin

**DOI:** 10.1007/s10811-017-1176-5

**Published:** 2017-06-10

**Authors:** Martin Sterner, Mauricio Sodré Ribeiro, Fredrik Gröndahl, Ulrica Edlund

**Affiliations:** 1 Fiber and Polymer Technology, KTH Royal Institute of Technology, Teknikringen 56, 100 44 Stockholm, SE Sweden; 2 Industrial Ecology, Department of Sustainable Development, Environmental Science and Engineering (SEED), KTH Royal Institute of Technology, Teknikringen 34, 100 44 Stockholm, SE Sweden

**Keywords:** Extraction, Fractionation, Alginate, *Saccharina latissima*, Phaeophyceae, Chelation, Ion exchange resin

## Abstract

A new approach to process *Saccharina latissima* algal biomass was developed using sodium citrate and a polyvalent cation-specific resin to sequentially extract the alginate into several usable fractions. The fractionation was performed in a cyclic manner, utilizing a stepwise removal of the native polyvalent ions present in the algae to isolate fractions of alginate with different solubility in the presence of these ions. Sodium citrate was used in different concentrations in the extraction solution to remove polyvalent cations to adjust the alginate liberation while AMBERLITE IRC718 resin was added to further remove these ions and regenerate the extraction solution. Alginate was recovered by acid precipitation and analyzed for its uronic acid composition and molecular weight, and the carbohydrate compositions of the insoluble and soluble parts of the algal biomass residue were determined. Finally, the fractionation method was assessed with a life cycle analysis to determine the energy and water efficiency as well as the greenhouse gas emissions and the results were compared to conventional alkaline extraction. The results indicate that the energy and water use as well as the emissions are considerably lower for the cyclic extraction in comparison with the conventional methods.

**Electronic supplementary material:**

The online version of this article (doi:10.1007/s10811-017-1176-5) contains supplementary material, which is available to authorized users.

## Introduction

The prospect of a large-scale aquaculture expansion at sea has a potential to generate large volumes of new biomass on so far unutilized spaces. The cultivation of sea-based crops comes with the benefit that it has no need for fertilization and irrigation. The production can give a high yield per cultivated area. New techniques to automatize the cultivation of fast-growing algae are developed to convert it to an industrial scale business, but to further pave the way for an algae-based industry, it is beneficial to also optimize and broaden the use of the crops. The search for a more complete utilization of biomass with fractionation techniques and valorization of the recovered components is referred to as biorefining (Kamm and Kamm 2004). The fermentable fraction with little other commercial value can be converted to biogas or ethanol, while more valuable components can be utilized for food, feed, and advanced material purposes.

The brown algal species *Saccharina latissima* thrives in the wild on the Swedish west coast, where a large-scale cultivation plant has been established (Pechsiri et al. 2016). *Saccharina latissima* grows quickly and generates biomass with anticipated high potential for biorefining (Nielsen et al. 2014; Vilg et al. 2015; Schiener et al. 2015). The main components of *S. latissima* are alginate, laminarin, mannitol, cellulose, proteins, and salts. Laminarin and mannitol are possible to ferment for energy purposes while alginate offers valuable viscosity and gelation properties (Draget et al. 2005; Schiener et al. 2016). Proteins may find use as feed and cellulose can be extracted in an unusually good quality due to the lack of lignin in brown algae.

Alginate is the one component of brown algae that so far has a commercial value, primarily in textile and food industries, but also in medical formulations (McHugh 2003; Andersen et al. 2012; Kraan 2012) where it is commonly used as viscosity modifier, for preparing gels or thin sheets (Aarstad 2013). The algal cell wall is primarily built up from alginate which constitutes approximately one fifth of the algal dry weight (Jard et al. 2013). Still, the remaining components far outweigh the alginate and it would enhance the commercial viability of the algal cultivation if the other components could find valuable uses.

One inherent problem, when mucilaginous components and other soluble components are to be simultaneously extracted from biomass, is that the large amount of extraction solution needed to handle the increase in viscosity increase from mucilage will prohibit a concentrated extraction of other components. Common ways to decrease viscosity during the extraction is to increase the temperature or, in the case of alginate extractions, to subject the biomass to an acid pretreatment that removes viscosity-mediating ions (Arvizu-Higuera et al. 1995). Both these methods degrade the extracted products (Haug et al. 1963; Hernández-Carmona et al. 1999a; Torres et al. 2007), and part of the solubles might as well end up in the acid of the pretreatment. This is indeed a dilemma hindering straightforward biorefining of brown algae where the need for high extracted concentration of the fermentable products laminarin and mannitol is in conflict with the need to maintain a low process concentration for a manageable viscosity.

We have previously studied the role of chelation in alginate extraction and discovered that if a strong chelating salt was used in the extraction solution, it interacted with the polyvalent cations and liberated more alginate (Sterner and Edlund 2016). When a very strong chelating salt was used, in this case sodium citrate, the same extraction solution could potentially be reused for repeated extractions and thus gradually increase the concentration of the other soluble components in it. Developing such an iterative extraction process may help overcome current obstacles in the realization of a biorefinery of brown algae with high efficacy and applicability. A challenge in such a process is to regenerate the used extraction solution and remove polyvalent cations. To develop successful and scalable fractionation methods of bio-derived polymers is a path forward to provide realistic renewable alternatives to synthetic polymers and open up new applications. A fractionation pathway that allows for separation of biopolymers into different grades would further increase their competitiveness. The fractionation of alginate by composition and molecular weight could make the products competitive with other polyelectrolytes with special product requirements.

Our aim was to take the concept of iterative extraction one step further by processing a large amount of algae with a controlled concentration of sodium citrate solution that is regenerated and cycled back so that only parts of the alginate is solubilized in each cycle. We hypothesized that the stepwise stripping of alginate from the biomass would keep the viscosity at a reasonable level and also fractionate the alginate into different qualities. Therefore, we developed a process for extraction of alginate from brown algae in a consecutive manner where regeneration of the spent extraction solution with an ion exchange resin that absorbs polyvalent cations allows for recycling of extraction liquid to minimize the required volume used. The recovered alginate fractions were characterized with respect to uronic acid composition and molecular weight. The ion exchange resin approach has hitherto not been applied in suchlike biorefining processes but is used in other industries such as in the purification of salt brines from polyvalent cations (Woodberry et al. 2005). Alginate yield and product properties have been examined using different extraction techniques, comparing method yields as a function of process parameters (Nishide et al. 1987; Wedlock et al. 1987; Ahmad et al. 1993; Hernández-Carmona et al. 1999b; McHugh et al. 2001; Vauchel et al. 2008; Sterner and Edlund 2016; Mazumder et al. 2016). In Haug and Smidsrød (1965), pre-extracted alginate was fractionated into two fractions, soluble and insoluble, by the addition of calcium and magnesium salts. The process presented in this paper was developed with a different aim: having a series of low yield partial extractions and to consecutively fractionate the alginate product directly when liberated from the algal biomass.

A further aim was to assess the viability of proposed cyclic fractionations from an environmental perspective focusing on chemicals, water and energy use, and greenhouse gas emission. This assessment was compared to a process representing the conventional industrial extraction of alginate involving one-step extraction in a dilute sodium carbonate solution followed by acid precipitation for alginate recovery (McHugh 2003; Hernández-Carmona 2013),

## Experimental

### Materials

**Scheme 1:**
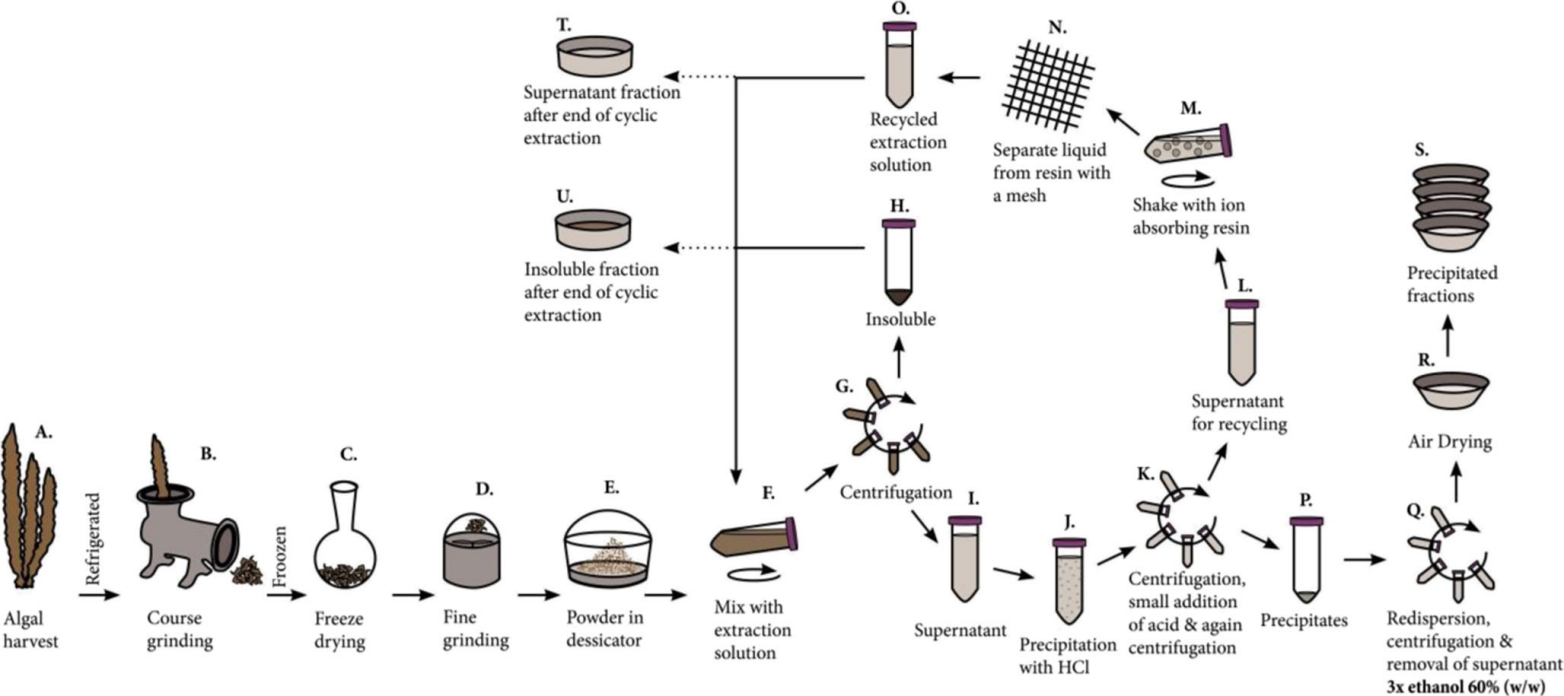
Experimental scheme for the cyclic fractionation of *Saccharina latissima* yielding several fractions precipitated by acid (T) and after the completion of the cycles as well supernatant fraction (U) and an insoluble fraction (V)

Samples of *S. latissima* were collected in November 11, 2013, at Ursholmen (N 58°50.124′, E 10°59.418), by the Sven Lovén Centre for Marine Science (University of Gothenburg) situated at the Swedish west coast (Scheme 1, step A.). The wet algae were refrigerated and then coarsely ground in a Bruker meat grinder equipped with three consecutive hole plates with hole diameters of 45, 5, and 2 mm, respectively (Scheme 1, step B.). The ground algae were frozen and kept at −20 °C until freeze dried (Scheme 1, step C.). Freeze-dried algae were ground into a fine powder in an OBH Nordica Coffee Mill, Type 2393 (Scheme 1, step D.) and stored in a desiccator with dry silica gel (Scheme 1, step V) until further use. The average particle diameter after grinding was 21 ± 33 μm assessed by light microscopy on 708 particles using a Leitz Ortholux II POL-BK, equipped with a Leica DC 300 digital camera.

Alginic acid from *Macrocystis pyrifera*, sodium citrate dihydrate ACS reagent ≥99%, sodium hydroxide ≥97% ACS reagent, hydrochloric acid 37% ACS reagent, AMBERLITE IRC718, L-(+)-arabinose ≥99, D-(+)-glucose ≥99.5%, D-(+)-mannose ≥99%, D-(+)-galactose ≥99%, D-(+)-xylose ≥99%, and D-(+)-fucose ≥99% were from Sigma Aldrich. D-(-)-mannitol purum was purchased from KEBO. Deuterium oxide (D_2_O) (DLM-4-100) 99.9% was from Cambridge Isotope Laboratories, Inc. Sulfuric acid 72% (*w*/*w*) was from Labservice. Ethanol 96% (*v*/*v*) was from VWR.

### Main extraction procedure

The preparation of the powdered algal sample followed by a cyclic fractionation procedure is schematically illustrated in Scheme 1. Duplicate samples were prepared for every extraction setting.

#### Start of fractionation

Solutions of sodium citrate (Na_3_C_6_H_5_O_7_) were prepared in three different concentrations: 0.01, 0.02, and 0.05 M, respectively, to be used as the extraction solution. Samples were prepared in 50-mL Falcon tubes to which 2.0 g algae powder and 38 g of sodium citrate solution were added followed by immediate mixing to homogenously disperse the algal powder.

#### The fractionation cycle

Falcon tubes were fixed horizontally on a shaking board with a shake frequency of 300 rpm and shake radius of 0.5 cm and left for 1 h at room temperature (Scheme 1, step F).

After extraction, each tube was centrifuged for 10 min at 3850*×g* (Scheme 1, step G). The formed pellet was manually crushed and centrifuged with the same setting again, a technique that gave more equal packing of the insoluble pellets than without the double centrifugation. After centrifugation, the supernatant was separated from the insoluble pellet and transferred to another 50-mL Falcon tube (Scheme 1, steps H and I). Hydrochloric acid (10 M) was then added to the supernatant, to give a pH of 1.0 ± 0.2 and allowed to precipitate for 1 h (Scheme 1, step J.). The solution with precipitated material was centrifuged for 5 min 2680*×g*. The precipitate pellet was then separated from the supernatant and dispersed in hydrochloric acid (pH 1.50) in the volume ratio 1:1 and centrifuged again with the same settings (Scheme 1, step K.). The supernatant from the two consecutive centrifugations was separated from the precipitate pellet and transferred to a 50-mL Falcon tube (Scheme 1, steps L and P). The extraction solution was purified for recycling by mixing it with the ion exchange resin beads, AMBERLITE IRC718, which is selective for polyvalent cations (Scheme 1, step M). The beads (5 g dry weight) were pre-wetted with 11.5 g extraction solution which was approximately the amount of liquid that could reside in the resin beads. The recycling was performed for 16 h in Falcon tubes fixed horizontally on a shaking board with a shake frequency of 150 rpm and shake radius of 0.5 cm. An adequate amount of sodium hydroxide (10 M) was added to allow the solution to become close to pH neutral after completed recycling, meaning the removal of cations from the solutions. Then, the resin beads were removed by filtration through a stainless steel wire (mesh 150) and stored in refrigerator for use in the next cycle in the fractionation (Scheme 1, step N). The recycled supernatant fraction (Scheme 1, step O) and the insoluble material fraction (Scheme 1, step H) was finally mixed again which was the start of the next extraction cycle (Scheme 1, step F). For every extraction, a reference sample duplicate was made in which the part of the extraction solution recycling was omitted (Scheme 1, step M and N). The reason to add the reference was to test to what extent the recycling enabled more alginate to be extracted for every cycle in the extraction solution and to be able to compare the extracted products with and without recycling.

#### Fractions from each cycle

The precipitates formed by the addition of hydrochloric acid (Scheme 1, step P) were washed by adding 45 mL of 60% (*w*/*w*) ethanol solutions, followed by shaking, centrifugation, removing the supernatant, and then adding another aliquot of washing solution (Scheme 1, step Q). Three washing cycles were performed with shaking at 150 rpm and shake radius of 0.5 cm. The shaking times were approximately 1, 16, and 1 h for the first, second, and last washing round, respectively. During washing, the samples were centrifuged for 5 min at 2680*×g*. The purified precipitate samples were transferred to weighted alumina cups in which they were dried under an airflow at room temperature, a process which took approximately a day (Scheme 1, step R.). The dry sample material in each cup (Scheme 1, step S) was then weighed and stored dry.

#### Final fractions

The number of extraction cycles that was run for each of the studied extraction solution concentrations was determined by the weight of the precipitated fraction. The extraction was stopped when it was no longer expected that the next fraction would weigh more than 100 mg by interpolation of the so far retrieved extraction weight data. After the final cycle, the remaining recycled supernatant and the remaining fraction containing the insoluble material were dried in weighted petri dishes (Scheme 1, steps T and U). The drying was performed under airflow at room temperature for a day.

### Characterization

#### Carbohydrate analysis

Carbohydrate analysis was performed via high-performance anion exchange chromatography (HPAEC). Two analyses were performed (1) a modified version of the standard SCAN-CM 71:09 (SCAN-CM 71:09 2009) and (2) a milder hydrolysis performed at 70 °C for 24 h.

##### Modified version of SCAN-CM 71:09

Samples were analyzed both from the precipitate fractions that was generated in each cycle in the extraction procedure (Scheme 1, step R.) and for the two fractions that were generated by the supernatant and insoluble material that was recovered at the end of the extraction (Scheme 1, steps T and U). A sample from each fraction was subjected to acid hydrolysis. For precipitated, supernatant, and insoluble material approximately 20, 600, and 300 mg of dry sample was used respectively for the hydrolysis. As a first step in the hydrolysis process, each sample was soaked in 3 mL 72% (*w*/*w*) of sulfuric acid while put in a desiccator under vacuum atmosphere for 80 min. The samples were manually grinded with a glass rod both directly after addition of acid and again during a temporary release of the vacuum. The second step in the hydrolysis procedure was to dilute the samples with 84 mL water, so that the sulfuric acid concentration reached 4% (*w*/*w*), followed by heating to approximately 125 °C for 1.5 h. The samples were hydrolyzed in 100-mL Pyrex flasks and heating was performed in a laboratory autoclave.

The carbohydrate compositions of the hydrolyzed samples were determined using a high-performance anion exchange chromatograph (Dionex, USA) equipped with a pulsed amperometric detector (HPAEC-PAD, Dionex ICS-3000) and CarboPac PA1 column (4 × 250 mm), using Milli-Q water and solutions of sodium hydroxide and sodium acetate. The eluent was pumped at 1.5 mL min^−1^ with a program starting with 0.10 M sodium hydroxide and increasing to 0.16 M sodium hydroxide with 0.19 M sodium acetate during the run.

The data were processed with Chromeleon 7.1 software. The carbohydrate standards used for calibration were mannitol, fucose, arabinose, galactose, glucose, xylose, mannose, and commercial alginate with a determined mannuronic/guluronic acid composition. The composition of the reference alginate was determined by NMR, and the reference alginate was hydrolyzed in the same way as the sample material.

##### Uronic acid analysis after milder hydrolysis

The precipitate fractions that were generated in each cycle in the extraction procedure (Scheme 1, step R) were hydrolyzed according to a milder protocol which only converted parts of the alginic acid into uronic acid monomers. For this hydrolysis, approximately 2 mg of sample was dissolved in 1 mL sodium hydroxide (0.005 M) in 2.5-mL glass vials. To the vials, 0.5 mL sulfuric acid 12% (*w*/*w*) was added followed by quick capping and shaking. The vials were shaken in standing position at 70 °C for 24 h with a shake frequency of 150 rpm and shake radius of 1.5 cm. The hydrolyzed sample was measured with the same chromatograph and settings as in the “Modified version of SCAN-CM 71:09” section but with the difference that the chromatograph eluent program ramped up quicker when increasing from 0.10 M sodium hydroxide to 0.16 M sodium hydroxide with 0.19 M sodium acetate during the run. A reference sample containing commercial alginate with a determined mannuronic/guluronic acid composition was run in parallel. The composition of the reference alginate was determined by NMR, and the reference alginate was hydrolyzed in the same way as the sample material.

#### Nuclear magnetic resonance

^1^H–NMR was used to determine the uronic acid composition of the alginate in the precipitated fraction (Scheme 1, step R). Samples were dissolved in water, and the pH was adjusted to 3 via small additions of dilute sodium hydroxide or hydrochloric acid. Then, samples were heated to 100 °C for 1 h. After cooling, the solutions were neutralized to pH 7 with sodium hydroxide, stirred until the alginate was completely dissolved, and then left to dry under an airflow at room temperature for approximately 1 day. Samples of approximately 2% dried material were dissolved in deuterium oxide (D_2_O) and transferred to NMR tubes with 5-mm diameters. ^1^H–NMR spectra were recorded at 25 °C at 500 MHz on a Bruker DMX-500 NMR spectrometer. MestReNova software was used for data acquisition.

**Fig. 1:**
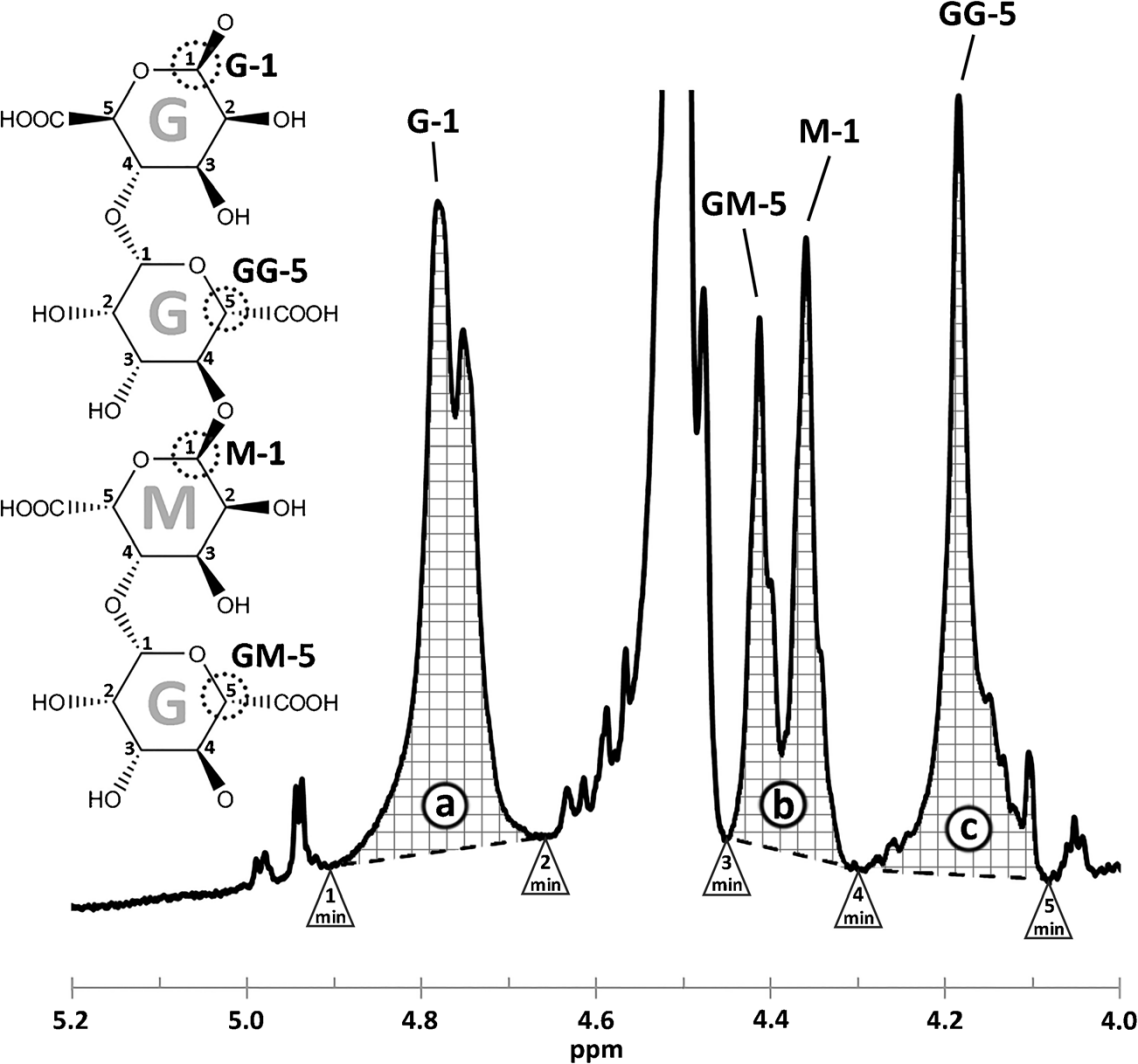
Areas of peaks **a**, **b**, and **c** in the ^1^H–NMR spectrum of extracted alginate showing the five minimum points in between in which the areas are calculated. Peak assignments to uronic acids and uronic acid dyads in the spectrum are indicated. The positions of the peak-generating hydrogen are outlined in an alginate sequence of GGMG

Equation 1 was used to determine the share of G and Eq. 2 to determine the share of GG dyads from the peak areas of a, b, and c in the ^1^H NMR spectra in Fig. 1 (Grasdalen et al. 1979). The areas a, b, and c were calculated after first smoothing the NMR curve by a centered simple moving average method to limit the impact of noise and differences in spectral resolution. The period for the moving average was 31 data points which approximately equaled 0.01 ppm. After smoothening, five minimum points were calculated in the intervals: 1 (5.13–5.07 ppm), 2 (4.90–4.84 ppm), 3 (4.68–4.62 ppm), 4 (4.52–4.46 pm), and 5 (4.32–4.26 ppm). The baseline and area delimiter for peak a was drawn between minimum 1 and 2; for peak b, the same was done between peak 3 and 4; and for peak c, it was done between 4 and 5.

1 $$ \mathrm{Share}\ \mathrm{of}\ \mathrm{G}=\frac{a}{b+ c} $$

2 $$ \mathrm{Share}\ \mathrm{of}\ \mathrm{GG}=\frac{c}{b+ c} $$

#### Size exclusion analysis

An estimate of the molecular weight distribution of the precipitated samples (Scheme 1, step R) was measured using a Dionex Ultimate-3000 HPLC system (Dionex, USA). The samples were prepared in the concentration 2 mg mL^−1^ dissolved in dilute sodium hydroxide (0.005 M) and then filtered through 0.2-μm Nylon filters. Sodium hydroxide (0.010 M) was used as the eluent, elution rate was 1 mL min^−1^, and the temperature kept in the system was 40 °C. Three PSS Suprema columns were used in series all with the dimensions 300 × 8 mm, with 10 μm particle size, and with the pore sizes of 30, 1000, and 1000 Å. A Waters-410 refractive index detector (Waters, USA) was used for measuring the chromatographic data. A set of pullulan polymers having molecular weights ranging from 342 to 708,000 g mol^−1^ was used for calibration. The data were processed with Chromeleon 7.1 software.

#### pH measurements

A VWR SympHony SB70P pH meter equipped with a Hamilton Biotrode electrode was used to measure the pH of the extraction solutions before and after extraction. CertiPur disodium hydrogen phosphate/potassium dihydrogen phosphate (pH 7.0) and CertiPur potassium hydrogen phthalate (pH 4.01) solutions from Merck were used for calibration.

#### Viscosity measurement

The viscosities of extraction solutions (the separated soluble extracts, Scheme 1, I) were assessed using a SCHOTT capillary viscometer (model 525 23) with a capillary diameter of 1.36 mm. Lauda iVisc measurement equipment was used for the automated viscosity measurements with Lauda iVisc software and a Lauda ECO Silver thermostat to keep the temperature at 25 °C.

#### Chemicals, water, and energy assessment

An assessment was done by quantifying the usage of chemicals, water, and process energy to produce 1 kg of alginate from dried *S. latissima* algae. This was done for the three processes presented in this paper and compared to the one-step sodium carbonate method assessed by Langlois et al. (2012) and selected as a reference study as it represents the extraction process widely used in the alginate industry (Hernandez-Carmona 2013). While there are a number of steps associated with sodium alginate production from algae, harvest, drying, grinding, chemical pretreatments, extraction, conversion to sodium alginate, and drying of product, the focus of the assessment herein was only on the extraction step. To be able to compare the processes, the maximum amount of extractable alginate was fixed to the maximum in our extraction (22.8%), also for the biomass of Langlois et al. (2012). The assessment was done in the life cycle perspective, following the ISO 14044:2006 guideline (International Organization for Standardization (ISO) 2006). The life cycle inventory was based on Ecoinvent 3.1 database (www.ecoinvent.org) and SimaPro 8.1.1.16 software. The Ecoinvent database provides process data for a broad range of products such as chemicals, energy supplies, and transportation (Steubing et al. 2016). The cyclic extraction presented in this paper used sodium citrate and ion exchange resin in the process and was compared to Langlois et al.’s (2012) assessment. In their work, the data was obtained from a pilot-scale experiment with direct measurement of materials and energy and in this work by measuring the materials use in the recycling loop, i.e., G, H, I, J, K, L, M, N, O, and P (Scheme 1), for all cycles. To estimate the energy use for the cyclic extraction, an upscaling scenario was modeled and performed using data from semi-industrial equipment for the centrifugation, mixing, and pumping. The industrial centrifuge model was a horizontal version, model PNX-418, made by Kingreat. A homogenizer, model JMC-500L, made by SINAIKA was employed in the assessment to represent an industrial blender that keep steering the solution during extraction and recovery in resin. For solution transport, an industrial liquid pump Hitachi, model TM-60L, was considered.

For this assessment, the inventory data for the production of all the inputs to the system were sourced from the Ecoinvent database 3.1 (Wernet et al. 2016). Assessment input data employed in both extraction methods were electricity from the Swedish grid, hydrochloric acid (30% solution), and freshwater (decarbonized water for industrial use). Unique input parameters for our process were sodium hydroxide (50% solution), sodium citrate, and cation selective resin (assumed lifespan of a minimum of 500 extractions). Unique input parameters for the Langlois et al. (2012) assessment were soda ash (sodium carbonate) and heat sourced by natural gas.

Due to the lack of specific data for sodium citrate in the Ecoinvent database, we considered the citric acid and sodium hydroxide as the input materials for the in situ production of sodium citrate. A similar approach was considered to evaluate the employed AMBERLITE resin; in this case, a generic cation selective resin in the database was used as proxy data (Wernet et al. 2016).

## Results and discussion

*Saccharina latissima* has a great potential to serve both as a source of carbohydrates for fermentation and a source of high viscous alginate with application for food and cosmetic products (Hernández-Carmona et al. 1999) as well as for textile industry (McHugh 1987). The value of alginate decreases if the component is degraded because high molecular weight is a key property for its applications. We previously showed that sodium citrate is a potent agent for extraction of alginate at neutral pH due to its high chelation strength at neutral conditions (Sterner and Edlund 2016). In this paper, we utilize the benefits of neutral extraction and extend the extraction into several cycles with reuse of the extraction solution. The advantage of extracting biomass in a cyclic manner with several consecutive extractions is that more biomass can be processed with the same extraction solution without experiencing problems with high viscosity. As a result, a higher concentration of the soluble carbohydrates can be retrieved which is beneficial for fermentation purposes. Another possible benefit of a cyclic extraction process is that the alginate could get fractionated into different qualities that have different properties, and thus, the value of the product could be increased.

### Extraction yields

Three different cyclic extractions were performed where the concentration of sodium citrate (Na_3_C_6_H_5_O_7_) in the extraction solution was set to 0.01, 0.02, or 0.05 M, respectively. The number of extraction cycles was 9, 7, and 5 for the concentrations 0.01, 0.02, and 0.05 M, respectively. Each extraction started with 2 g algae and 38 g extraction solution, and pH was adjusted to 7 ± 0.2 at the start of every extraction cycle.

**Fig. 2:**
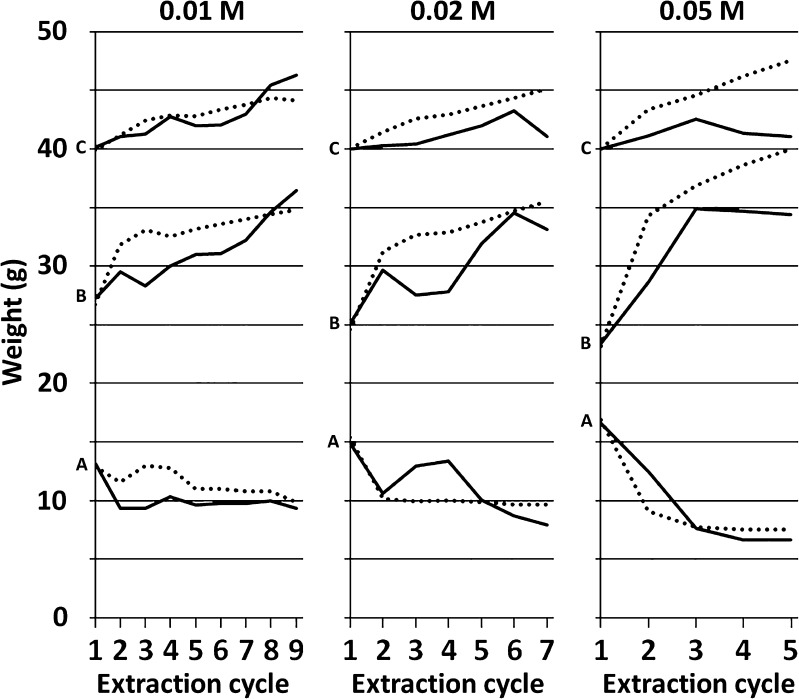
Average weight of the extraction fractions for each extraction cycle: (*solid line*) with and (*dotted line*) without polyvalent cation removal from the extraction solution. **a** Insoluble pellet after centrifugation. **b** Supernatant after centrifugation. **c** Total weight of during extraction. From left to right: extractions with 0.01, 0.02, and 0.05 M sodium citrate

The amount of extraction solution increased somewhat during the course of the extraction due to the addition of acid and base for the acid precipitation and neutralization in every cycle. The amount of hydrochloric acid (10 M) used for precipitation of alginate for every cycle in the process was 0.3, 0.4, and 0.65 mL for the sodium citrate concentrations 0.01, 0.02, and 0.05 M, respectively, resulting in a pH during precipitation in the range of 1.0 ± 0.2. The neutralization of the supernatant extraction solution to be recycled was partly an effect of a pH rise due to absorption of polyvalent ions in the ion exchange resin which released sodium ions that increase pH. Accordingly, no or little sodium hydroxide is needed to be added in the initial cycles, while in the end, when only little polyvalent ions were left in the solution, equal amounts of sodium hydroxide (10 M) were needed to be added as the previously added hydrochloric acid (10 M). The removal of the precipitated alginic acid (Scheme 1, step P) in every cycle also changed the volume of extraction solution; this was however compensated for by the addition of extra liquid (dilute HCl) in Scheme 1, step K. Due to changes in the algal biomass and differences in the viscosity rise of the extraction solution, the insoluble material fraction was also differently densely packed after the centrifugation (Scheme 1, step H). The general trend during the course of extraction was that the insoluble material got more densely packed so that a greater share of the total extraction solution ended up in the supernatant fraction, and also that the total amount of extraction solution increased. Denser packing of the insoluble material was also observed with higher concentration of sodium citrate in the extraction solution. In comparison, the reference extractions (without recycling of the extraction solution) gave a more rapid build-up of dense packing of the insoluble fraction which led to that the ratio of the supernatant to insoluble fraction increased sooner in that process. Also, in the case of the reference samples, the total amount of extraction solution increased quicker due to the lack of pH neutralization arising from the use of ion exchange resins; hence, more sodium hydroxide was required to compensate for the addition of hydrochloric acid in the precipitation step. The average weights of insoluble fraction, supernatant, and the total weights of both fractions combined are summarized in Fig. 2.

If there is a high supernatant to insoluble ratio after the centrifugation step (Scheme 1, step G), it will be possible to recover more alginate by precipitation from the supernatant (Scheme 1, step J). The average supernatant/insoluble ratio varies during the course of the extraction (Table 1), and the greatest difference is seen in the first cycles of the extraction, especially in the extractions when higher concentrations of sodium citrate are used.

**Table 1:** Proportions of extraction solution present in the supernatants after each extraction cycle

Extraction cycle		1	2	3	4	5	6	7	8	9
With polyvalent cation removal (% *w*/*w*)	0.01 M	68%	72%	69%	70%	74%	74%	75%	76%	79%
	0.02 M	63%	74%	68%	67%	76%	80%	81%		
	0.05 M	58%	70%	82%	84%	84%				
Without polyvalent cation removal (% *w*/*w*)	0.01 M	67%	77%	78%	76%	78%	77%	78%	78%	79%
	0.02 M	61%	75%	77%	77%	77%	78%	79%		
	0.05 M	58%	79%	83%	84%	84%				

An understanding of the distribution of fractions in the wet state enables better interpretation of the data from the dried precipitated fractions. Since there is always a fair share of alginate that goes into the next cycle, each fraction except the first one is in one sense a mix of the alginate from the previous cycle and freshly liberated alginate from the current cycle.

**Fig. 3:**
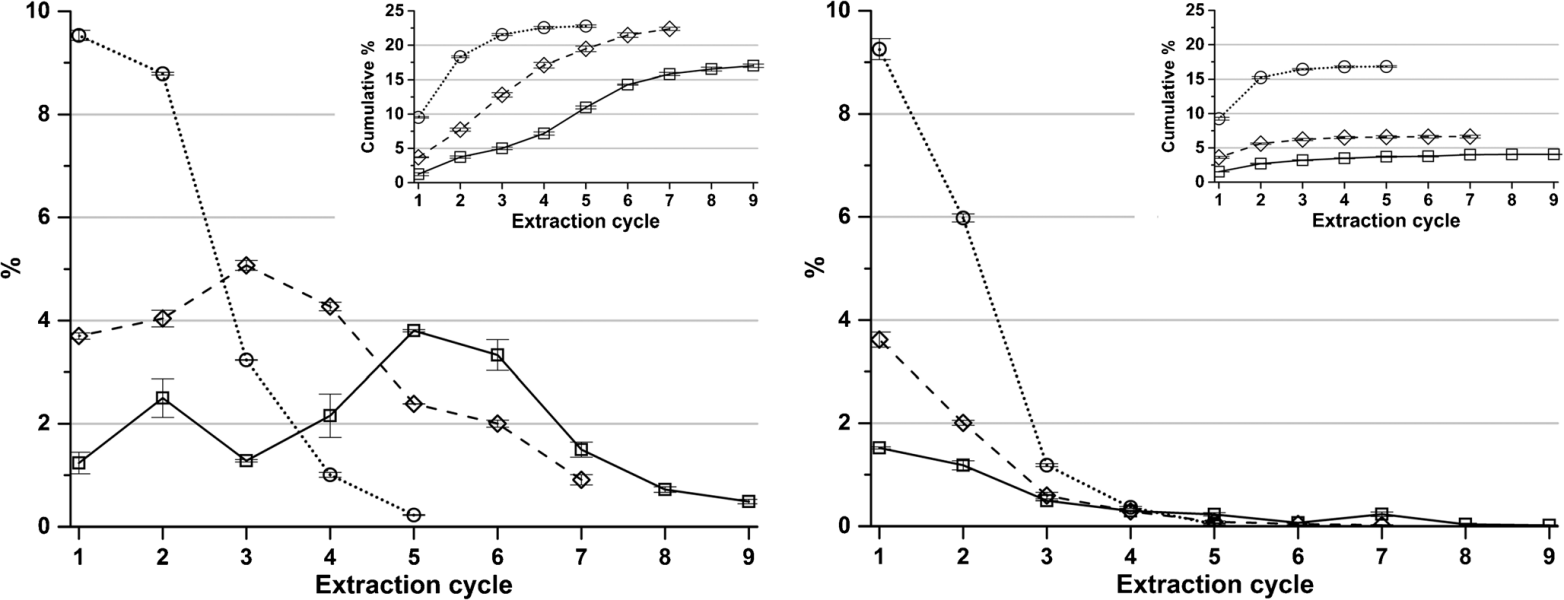
Yield in %(*w*/*w*) of starting dry algae, for the precipitated fractions for each extraction at sodium citrate concentrations (*square*) 0.01 M, (*diamond*) 0.02 M, and (*circle*) 0.05 M with (left graph) and without (right graph) polyvalent cation removal from the extraction solution. *Error bars* represent standard deviation of two independent replicates. Cumulative %(*w*/*w*) yields are given in the upper right insets

The yield of extracted amount is higher in extractions where ion exchange resin was used to recycle the extraction solution compared to the reference extraction without polyvalent cation removal from the extraction solution (Fig. 3). With higher concentrations of sodium citrate in the extraction solution, more alginate is dissolved per cycle and thus fewer cycles are needed to extract the greater part of the alginate. Interestingly, the precipitated amount is not the highest in the first extraction cycles but instead peaks in the later cycles in extractions using the two lower concentrations of sodium citrate. It was clearly observed that the extraction solution viscosity peaked in the first cycle, most likely since it had the highest amount of liberated polyvalent cations. To investigate this, the viscosities of the separated soluble extracts (Scheme 1, I) were measured for (i) a full one-step extraction with 0.2 M sodium citrate and (ii) after the first step of a cyclic extraction with 0.02 M sodium citrate. The viscosity was significantly lower in the cyclic extraction compared to a full one-step extraction. The full one-step extraction gave a viscosity of 70 mm^2^ s^−1^ in the extraction solution, while the corresponding viscosity was only 6.2 mm^2^ s^−1^ after the first cycle of the cyclic extraction.

In Fig. 3, one of the samples in the extraction with 0.01 M sodium citrate contained 2.06 g dry algae due to an experimental error compared to 2.00 ± 0.01 for the rest of the samples which could explain the greater difference in yield for those double samples.

A reasonable explanation to why the precipitated fraction peaked in the later cycles is that the extraction solution initially got saturated with polyvalent cations from the algae. A few cycles of extraction solution recycling allowed for the adsorption of these polyvalent cations and decreased their concentration enough to allow the bulk of alginate to be extracted. In the case of extraction with 0.05 M sodium citrate, the highest precipitated amount was instead recovered in the first cycle, indicating that this concentration of sodium citrate was high enough to liberate the major part of alginate already from the start, leaving less to be extracted in the later cycles and reducing the effect of extraction solution recycling. This is sustained by the relatively low difference between extraction with and without polyvalent cation removal from the extraction solution of the extraction solution. There was an increase of solution ionic strength due to build-up of sodium chloride from repeated acid precipitation followed by neutralization. According to our previous study (Sterner and Edlund 2016), the increased ionic strength should decrease the polyvalent cation affinity of the sodium citrate while the pure addition of sodium chloride should give a small but positive effect on the alginate extraction. The current work with the use of polyvalent cation-specific resin shows that alginate can be extracted in the presence of high sodium chloride concentration as long as the polyvalent cations are removed, and that it does not significantly aid the extraction if those are still present.

The average total yield of all the precipitated material is listed in Table 2. Only a small part of the total alginate can be extracted in a single extraction cycle if a low concentration of the chelating salt sodium citrate is utilized which is consistent with the results of other studies. In Mazumder et al. (2016), an incomplete alginate extraction is seen when a lower concentration of the chelating salt sodium carbonate is utilized. In Sterner and Edlund (2016), an incomplete alginate extraction is instead seen when a high concentration of salt is used in the extraction solution but the chosen salt has low chelation strength.

**Table 2:** Total yield (% *w*/*w*) of extracted precipitate material

Sodium citrate concentration	0.01 M	0.02 M	0.05 M
Extraction with polyvalent cation removal	17.0 ± 0.2	22.4 ± 0.2	22.8 ± 0.2
Extraction without polyvalent cation removal	4.1 ± 0.1	6.6 ± 0.2	16.8 ± 0.1

There is a difference in the total weight of extracted material between the extraction at 0.01 M sodium citrate concentration and the other two concentrations. This indicates that alginate extraction is more efficient at higher sodium citrate concentrations. Another contributing factor may be that the precipitate product was recovered in many smaller fractions which could allow for more mass losses in the purification step (Scheme 1, step Q). A higher pH rise in the extraction solution recycling step (Scheme 1, step M) at 0.01 M sodium citrate concentration could possibly also degrade some of the soluble components and make them easier to remove in the purification step (Scheme 1, step Q). The average pH after the extraction solution recycling (Scheme 1, step M) is found in Table 3.

**Table 3:** Average pH after the extraction solution recycling

Extraction cycle	1	2	3	4	5	6	7	8	9
0.01 M	10.5	10	9	7	7	6	6	5.5	5.5
0.02 M	9.5	8.5	7	6.5	7	7	7		
0.05 M	9	6	5.5	6	7				

Polyvalent cations have the tendency to crosslink alginate and render it insoluble if the concentration is high enough. The minimum concentration of polyvalent cations required to mediate crosslinking of alginate is dependent on the uronic acid composition of alginate: with a higher ratio of mannuronic acid (M), alginate is more easily dissolved in the presence of polyvalent cations than when the guluronic acid (G) ratio is higher. The fact that the extraction is performed with a gradually decreasing concentration of polyvalent cations present for every extraction cycle could hence influence the extraction so that alginate with different M/G ratio is retrieved in the different cycles of extraction. The precipitated fractions (Scheme 1, step S) were examined with NMR and anion exchange chromatography to shed light on the compositional differences.

### Compositional analyses of the precipitated fractions

The precipitated fractions from the three cyclic extractions with different concentrations of sodium citrate were analyzed by NMR to determine the share of the two uronic acids, guluronic acid (G) and mannuronic acid (M), in the alginate (Fig. 1). The uronic acid compositions were calculated from the peak areas of a, b, and c in the ^1^H NMR spectra using Eqs. 1 and 2 as described in the “Nuclear magnetic resonance” section.

**Fig. 4:**
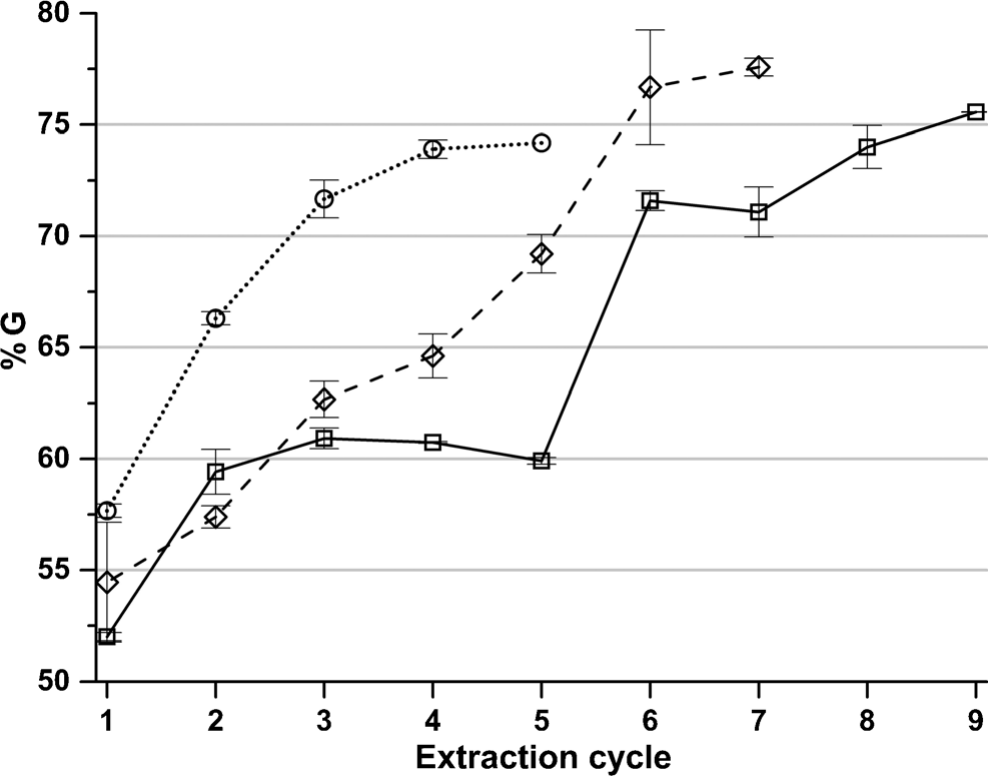
The content of G in the precipitated alginate in each extraction cycle for extractions with polyvalent cation removal at sodium citrate concentrations (*square*) 0.01 M, (*diamond*) 0.02 M, and (*circle*) 0.05 M. *Error bars* represent standard deviation of two independent replicates

Alginate fractions recovered in later cycles have a greater share of G, probably since alginate high in G is more likely to dissolve with lower concentrations of polyvalent cations present during the extraction. Since the ion exchange resin removes a portion of the polyvalent cations in every cycle, their concentration is gradually decreasing so that more alginate can be extracted. The G content in the precipitated alginate fraction in each extraction cycle is shown in Fig. 4. This can be compared with the results from Haug and Smidsrød (1965) showing that the fraction with a higher G content cannot be dissolved in the presence of calcium and magnesium.

**Fig. 5:**
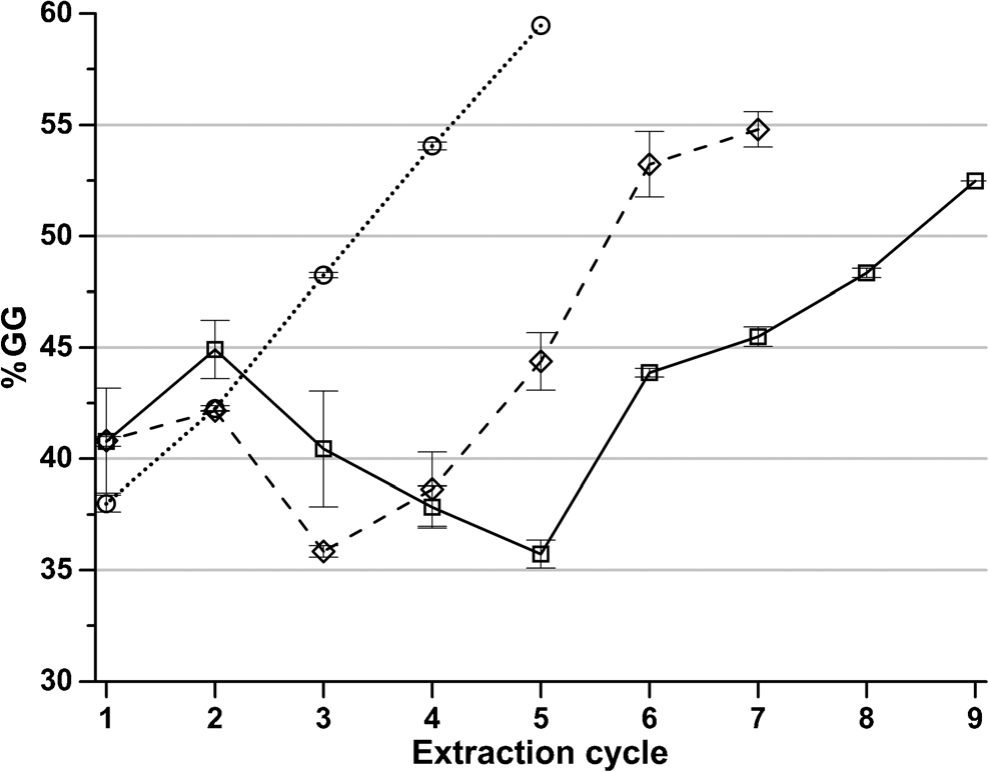
The content of GG dyads of the precipitated alginate in each extraction cycle for extractions with polyvalent cation removal at sodium citrate concentrations (*square*) 0.01 M, (*diamond*) 0.02 M, and (*circle*) 0.05 M. *Error bars* represent standard deviation of two independent replicates

Clearly, there is an increase in G content in alginate recovered in later cycles. A similar trend is seen when calculating the GG dyads, shown in Fig. 5, but rather than a rising trend from start to end, the G content initially increases followed by a decrease and then a major rise. An increase in G while GG decreases is not contradictory since the sequence distribution of M and G also need to be taken into account. If G is present in longer blocks in the alginate sequence; it becomes less soluble in the presence of polyvalent ions than if there are mostly repeating MG in the sequences (Draget et al. 2005).

The NMR pattern is changing during the course of the cyclic extraction. The most visible difference is that peak GM-5 is increasing while M-1 is decreasing. M-1 decreases with a decreasing amount of mannuronic acid. With increasing G content and decreasing M content, the probability of having GM segments increase, explaining the observed increase in intensity of the peak GM-5 peak. The nine NMR spectra of one of the samples of the extraction with 0.01 M sodium citrate are plotted in Online resource 1.

### Compositional analyses of the extracted fractions with hydrolysis and anion exchange chromatography

The precipitated fractions (Scheme 1, step S) gathered during the cyclic extraction as well as the remaining soluble and insoluble fractions after the end of extraction (Scheme 1, step T and U) were subjected to acid hydrolysis, and their carbohydrate compositions were analyzed with ion exchange chromatography (the “Carbohydrate analysis” section). Each sample was divided into two portions, and the portions were hydrolyzed in dilute sulfuric acid in two different ways: at a temperature of 125 °C for 1.5 h aiming to fully hydrolyze the carbohydrate and at 70 °C for 20 h to slowly hydrolyze the alginic acid and give a more reproducible measure of the uronic acid composition (the “Modified version of SCAN-CM 71:09” and “Uronic acid analysis after milder hydrolysis” sections). The samples differed in the amount of available supernatant at the end of the extraction since a non-equal portion of it was retained in the insoluble fraction and among the polyvalent cation-specific resins. There was also a difference in the amount of supernatant that was lost in the precipitated fractions. These differences were compensated for so that the sugar analysis of supernatant could be stated as the total extracted in mg g^−1^ dry algae. The calculated yields of soluble carbohydrates for the three cyclic processes with 0.01, 0.02, and 0.05 M sodium citrate were 78, 72, and 80%, respectively.

**Fig. 6:**
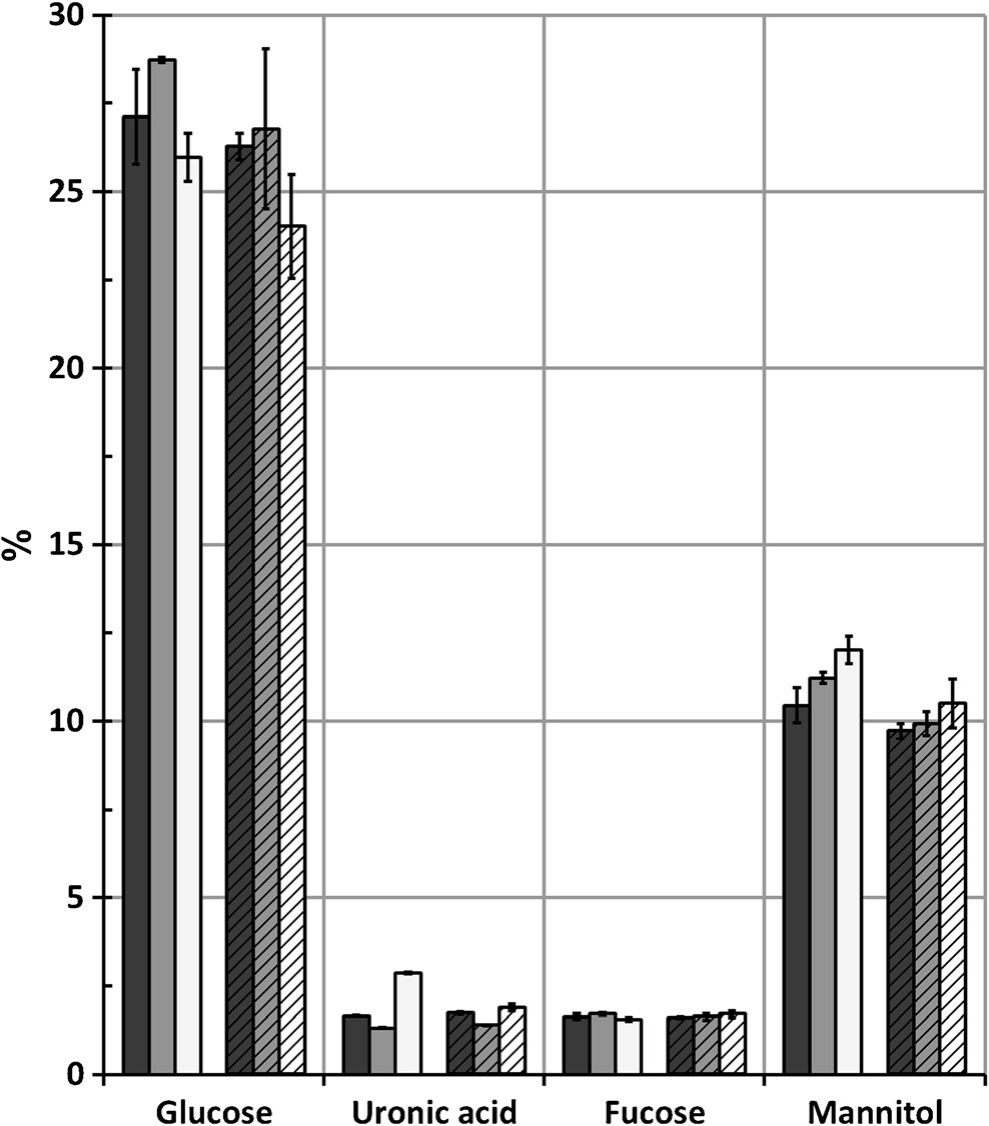
Carbohydrate composition in %(*w*/*w*) of the hydrolyzed supernatant fractions (fraction O, Scheme 1) for extraction with (filled) or without (striped) polyvalent cation removal from the extraction solution with sodium concentrations (*black square*) 0.01 M, (*gray square*) 0.02 M, and (*white square*) 0.05 M. *Error bars* represent standard deviation of two independent replicates

**Fig. 7:**
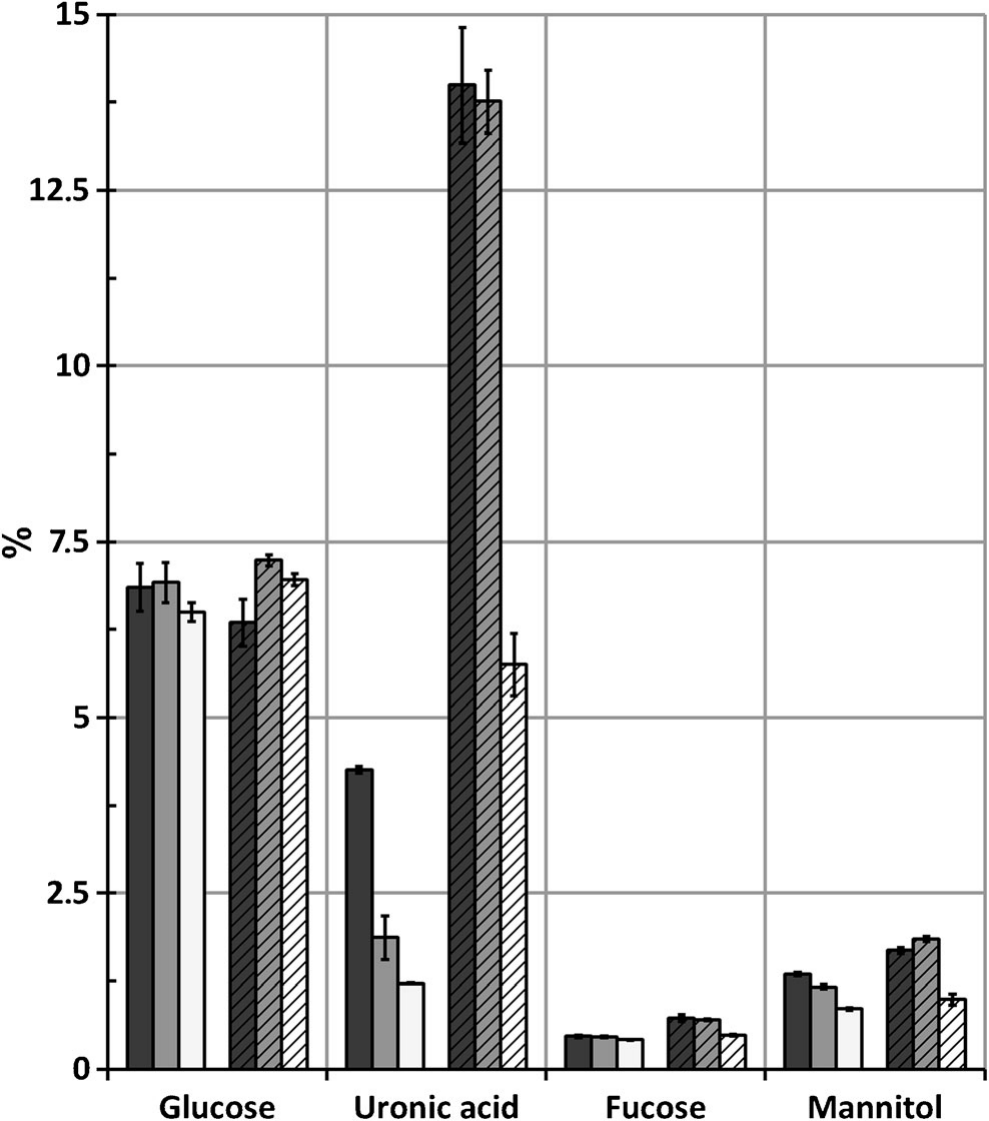
Carbohydrate composition in %(*w*/*w*) of the hydrolyzed insoluble fractions (fraction H, Scheme 1) for extraction with (filled) or without (striped) polyvalent cation removal from the extraction solution with sodium concentrations (*black square*) 0.01 M, (*gray square*) 0.02 M, and (*white square*) 0.05 M. *Error bars* represent standard deviation of independent replicates

The results from sugar analysis of supernatant and insoluble fractions (Scheme 1, step T and U) are shown in Figs. 6 and 7. Closely similar amounts of glucose, uronic acid, fucose, and mannitol are found in the remaining supernatant fraction after the end of the extraction, regardless of the sodium citrate concentrations in the extraction solutions and whether polyvalent cation-specific resins were applied or not. The glucose sugars originate mostly from laminarin, fucose from fucoidan, and uronic acids from alginate, while mannitol is a sugar alcohol in the algae (Pronina et al. 2012; Jard et al. 2013).

The minor amount of residual uronic acid in the soluble fractions verifies that precipitation has been effective in removing alginate from the supernatants. Glucose should origin from cellulose instead of laminarin in the insoluble fraction, while the uronic acids originate from alginate that was not dissolved during the extraction process. It must also be noted that approximately one tenth of the soluble fraction was dried together with the insoluble fraction (the presence of mannitol in the insoluble fraction is a clear indication), so the glucose will partly originate from laminarin and partly from cellulose. The benefit of extraction solution recycling is clearly seen in Fig. 7: significantly higher amounts of alginate remain unextracted in the reference extraction while very little alginate remains when polyvalent cation removal from the extraction solution is applied. With increasing concentrations of sodium citrate in the extraction solution, less alginate is found in the insoluble sample after the finished extraction, sustaining the result of the total amount of precipitated alginate given in Table 2.

The values of the carbohydrates are within published values compared to previous studies on *S. latissima* harvested at the same time of the year in Sweden and Scotland at the same latitude as the Swedish west coast, respectively. (Schiener et al. 2015; Sterner and Edlund 2016) (Table 4). Since about one tenth of the soluble material is incorporated in the insoluble fraction, it is estimated that about 2.5% of the glucose found in the insoluble fraction is laminarin.

**Table 4:** Comparison of approximate composition soluble and insoluble fractions (as % dry matter) found in the present study compared to related studies in literature

	Laminarin (soluble glucose)	Cellulose (insoluble glucose)	Mannitol	Fucose
Present study	30%	5%	10%	2%
Sterner and Edlund (2016)	25%	5%	10%	2%
Schiener et al. (2015)	15%	15%	20%	-

**Fig. 8:**
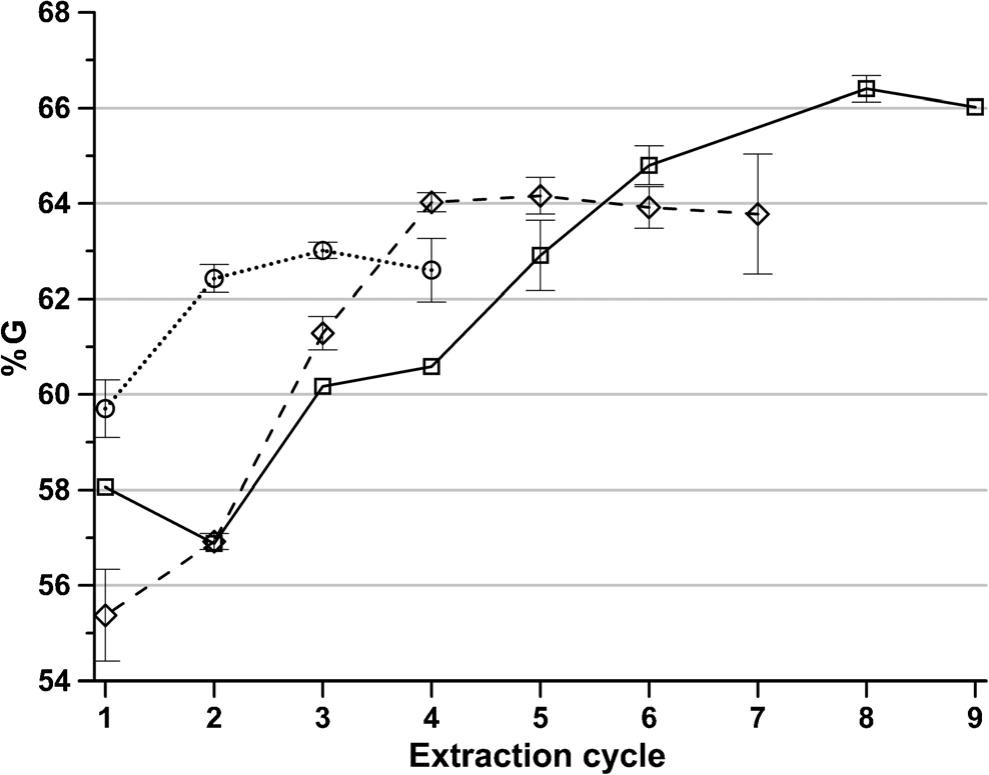
Guluronic acid (G) content of the precipitated fractions determined by anion exchange chromatography for extractions at sodium citrate concentrations (*square*) 0.01 M, (*diamond*) 0.02 M, and (*circle*) 0.05 M. *Error bar* represents standard deviation of two independent replicates

The precipitated fractions (Scheme 1, step S) were also analyzed with respect to their carbohydrate composition. The relative amount of uronic acids versus the other analyzed species (glucose, fucose and mannitol) after hydrolysis was in the range 90–99%. NMR data (Figs. 4 and 5), suggesting that the G content of alginate increased in the later extraction cycles, and to further test if this trend was true, the G content was also analyzed with anion exchange chromatography (Online resource 2). Indeed, an increase in G content can be seen but the results are not conclusive. A possible complication is the heterogeneous hydrolysis of the different samples which in combination with the fact that G is more prone to degradation than M gives room for significant experimental error. Another hydrolysis protocol was hence applied to ensure hydrolysis under more reproducible conditions (the “Uronic acid analysis after milder hydrolysis” section). The result from this analysis (Fig. 8) are much more clear-cut and reveal the same trend as anticipated in (Online resource 2). Anion exchange chromatography complemented the NMR analysis and independently showed the same trend of increasing G content with increased extraction cycles. The composition of the reference alginate, which acts as a scaling factor to the anion exchange chromatography measurements, was however determined by NMR and gave dependence between the two analyses. The accuracy of the anionic exchange chromatography assessment should be best when the uronic acid compositions of sample alginate and reference alginate are similar, and they behave more equal in the acid hydrolysis (G content of reference alginate was 58%). Values for anion exchange chromatography analyses of hydrolyzed precipitate samples with and without polyvalent cation removal are found in Online resource 3.

**Fig. 9:**
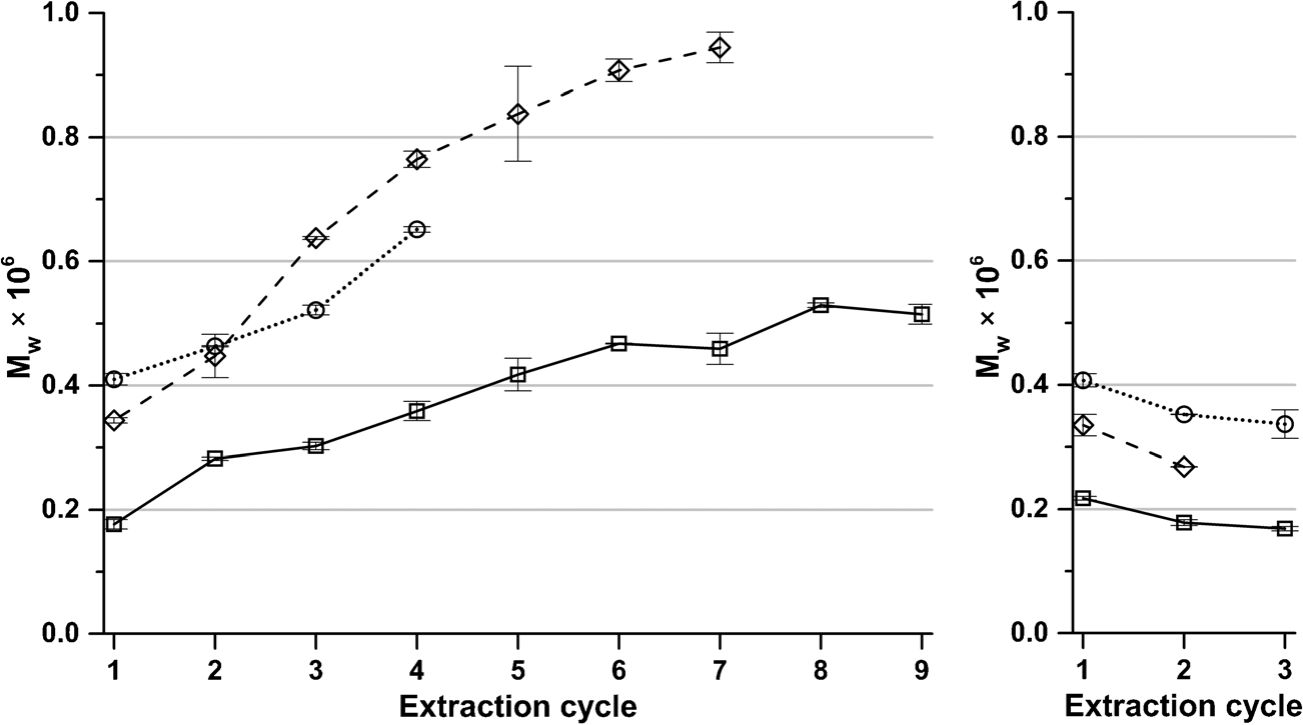
Average molecular weights (M_w_) of the precipitated fractions determined by SEC for extractions at sodium citrate concentrations (*square*) 0.01 M, (*diamond*) 0.02 M, and (*circle*) 0.05 M with (*left graph*) and without (*right graph*) polyvalent cation removal from the extraction solution. *Error bars* represent standard deviation of two independent replicates

The precipitated fractions were in addition analyzed using aqueous SEC (Fig. 9). With every cycle, there is an increase in the weight average molecular weight (M_w_) of the precipitate alginate. The values of M_w_ should however be treated with some precaution since the values are determined in relation to a reference weight substance (in this case pullulan) which differ in hydrodynamic volume from the charged polymer alginate. Still, it is clear that the molecular weights are increasing in every extraction cycle which implies that the molecular weight of alginate strongly influences its potential to be liberated with a given amount of polyvalent cations present during the extraction. Since the commercial value of alginate is strongly related to its molecular weight and the M/G ratio, the herein proposed cyclic extraction procedure may serve as a key enabling technology for a future biorefinery where various grades of alginate can be recovered from brown algae in a controlled fashion. Even though the M_w_ is not completely reliable, the higher values reported here are in comparison with literature values of ultra-high viscosity alginate. As evident from Fig. 9, the recovered alginate fractions have molecular weights as high as 0.9·10^6^ g mol^−1^ which can be compared to a reported value of 1.1 × 10^6^ g mol^−1^ for ultrahigh viscosity alginate (Storz et al. 2009). Two purchased commercial grade alginates were analyzed with SEC for comparison and shown to have an M_w_ of 0.1 × 10^6^ and 0.4 × 10^6^ g mol^−1^, respectively, being lower than the extracted alginate in this study. In the extractions done without cation removal from the extraction solution, M_w_ remained fairly constant but decreased slightly for each cycle. A net decrease in M_w_ could arise from the fact that several factors influence the slow liberation of alginate in an extraction solution saturated with polyvalent ions. Low M_w_ alginate with a uronic acid composition not favoring liberation (rich in G) or with a longer distance to migrate to the extraction medium would result in a slower extraction. The share of guluronic acid was indeed increasing slightly for these fractions during the progress of the extraction, as evident from the NMR analysis (the NMR spectra of the 3–4 first fractions extracted without polyvalent cation removal are shown in supplementary information as online resources 4 and 5). Based on the above, a more significant decrease of M_w_ would have been expected. However, we observed only a moderate decrease, which can be understood by considering that these fractions partly consist of liberated alginate from the previous cycle that remained trapped in the insoluble fraction and were hence not precipitated (Scheme 1, step H).

The nine SEC traces of one of the samples of the extraction with 0.01 M sodium citrate are plotted in Online resource 6.

### Chemical, water, and energy assessment

The assessment was conducted by evaluating and comparing the impacts on the environment regarding the chemical, water, and process energy needed for the three multistep cyclic extractions presented in this paper, to the more conventional one-step extraction assessed by Langlois et al. (2012). We compared the amount of freshwater and energy required to produce 1 kg of dried alginate. We also calculated and estimated the greenhouse gas potential, expressed in CO_2_-eq (UNFCCC 2008), for the processes. ReCiPe Midpoint method (Goedkoop et al. 2013) was used to calculate the CO_2_-eq. The comparison in terms of process energy, water requirement, and CO_2_-eq is found in Table 5.

**Table 5:** Comparison between the assessed processes for alginate extraction from *Saccharina latissima* and values of climate change (kg CO_2_-eq) contribution for the proposed alginate cyclic extraction

Parameters and impacts		Extraction procedures per kg of alginate			
		Reference	Present study		
		J. Langlois et al. (2012)	0.01 M	0.02 M	0.05 M
			Sodium citrate	Sodium citrate	Sodium citrate
Inputs	Energy (MJ)	172.5	28.69	16.96	11.88
	Freshwater used (L)	568.29	55.87	42.40	41.66
	Sodium citrate (kg)	–	0.29	0.44	1.08
	Sodium hydroxide (50%) (kg)	–	2.35	2.50	3.16
	Hydrochloric acid (30%) (kg)	9.40	3.57	3.80	4.80
	Sodium carbonate (kg)	4.51	–	–	–
Impact on climate change (kg CO_2_-eq)	Energy	5.45	0.31	0.19	0.14
	Water use	5.0 x 10^-3^	4.9 x 10^-4^	3.7 x 10^-4^	3.7 x 10^-4^
	Sodium citrate	–	2.21	3.36	8.25
	Sodium hydroxide (50%)	–	3.15	3.34	4.22
	Hydrochloric acid (30%)	7.62	2.90	3.08	3.89
	Cation selective resin	–	7.9 x 10^-4^	7.9 x 10^-4^	7.9 x 10^-4^
	Sodium carbonate	4.13	–	–	–
	Total	19.63	8.56	9.97	16.50

The energy use was about ten times higher for extraction conducted by Langlois et al. (2012) compared to the present study. The major reason for this is that their procedure involved heating to 50 °C followed by cooling. In the cyclic extraction procedure presented here, the required amount of energy decreases with an increase of sodium citrate concentration. This is explained by the lower amount of cycles needed with a higher sodium citrate concentration, which implies less stirring, centrifugation, and pumping.

The freshwater use was also about ten times higher for the Langlois et al. (2012) method. This is explained by the use of a lower concentration of processed algae, which is a requirement if all the alginate is to be extracted at once in a direct process, with reasonable viscosity. For the cyclic procedure, the yield is lower at a sodium citrate concentration 0.01 M, compared to extractions with 0.02 and 0.05 M sodium citrate, respectively. As a result, the water required is higher for the lowest concentration of sodium citrate, since the numbers are calculated for production of 1 kg of dry alginate.

The CO_2_-eq emission is about twice as high for the Langlois et al. (2012) extraction than for the cyclic extraction at the highest concentration of sodium citrate (0.05 M). The major contributing factor is the production of the chemicals, in particular the sodium citrate but also the sodium hydroxide and hydrochloric acid. The CO_2_-eq emissions for the used chemicals are listed in Table 5.

The chemicals involved are the biggest source of greenhouse gas emission, with far higher impact than the electricity used for processing. It can also be noted that the water and reusable cation selective ion exchange resins have an almost insignificant contribution for this impact. Notably, the regeneration of the resin is not included in the current assessment. As this regeneration step requires the use of acid followed by neutralization, some additional impact would be added if this step was included as well. Sodium citrate is used in lower amounts than hydrochloric acid and sodium hydroxide but has a higher impact per kilogram; this motivates the use of smaller amounts of sodium citrate in the cyclic process at the expense of higher usage of hydrochloric acid and sodium hydroxide. It must be stated that this assessment was only done for the extraction part and that some energy demanding steps are left out, for instance, the drying of the finished alginate product, but these steps should be equally energy demanding for both methods.

## Conclusions

A cyclic process with chelating salt and reuse of the extraction solution was developed to allow for stepwise recovery of alginate from *S. latissima*, thereby evading the otherwise concurrent viscosity build-up in conventional alginate extractions. Extractions with sodium citrate as chelator, operating at neutral pH, enabled consecutive extractions without degrading valuable algal components. Ion exchange resins effectively regenerated the extraction solution before the start of each new cycle by removing native ions released from the algal biomass. The cyclic process enabled the fractionation of alginate products with distinctly different properties both regarding molecular weight and uronic acid composition. More fractionation cycles increased the differentiation of the alginate product, giving an increase of guluronic acid from about 54 to 78% from the start to the end of the extraction with most cycles. The same trend was observed for the molecular weight (M_w_) which increased about 2.5 times reaching a value as high as 0.9 × 10^6^ g mol^−1^.

At the same time, the fact that alginate was extracted in a stepwise manner allowed for direct processing of more biomass at once since the viscosity rise due to alginate dissolution was limited. This allowed for a high concentration of the soluble carbohydrates, laminarin, and mannitol, giving a fraction that could be utilized for fermentation purposes. Our environmental assessment indicated that the energy and water use, as well as the greenhouse gas emissions to the atmosphere, to extract alginate were considerably lower for the cyclic process compared to a reference one-step process. The regeneration of the extraction solution allowed for effective extractions using low concentrations of sodium citrate, something positive in terms of greenhouse gas emission since sodium citrate had the highest impact per kilo of chemical. The environmental impact of processing energy was low compared to that of producing the involved chemicals which justified a more energy demanding extraction with more processing cycles but with less total chemical input.

As a concluding remark, this extraction technique worked well for alginate extractions from *S. latissima* both from a technical and environmental perspective. The generation of a concentrated fermentable carbohydrate fraction could benefit this process as well as the inherent fractionation of alginate that could favor high value applications with demand on molecular weight and uronic acid composition.

## Electronic supplementary material

ESM 1 (DOCX 244 kb)
