# Supplementary material for: Cyclic fractionation process for Saccharina latissima using aqueous chelator and ion exchange resin
Source: J Appl Phycol. 2017 Jun 10;29(6):3175–89. doi: 10.1007/s10811-017-1176-5 (PMC5705741; doi:10.1007/s10811-017-1176-5)
Supplement: Supplementary file 1 — (DOCX 244 kb) [file 10811_2017_1176_MOESM1_ESM.docx]

**Online resources**


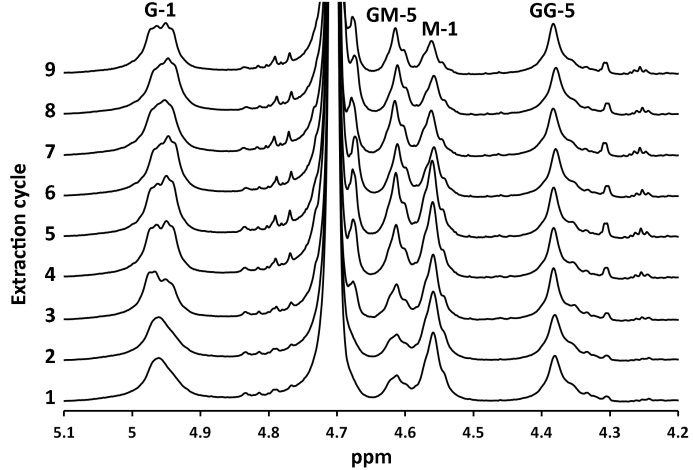


Online resource 1. NMR spectra of fractions from extraction cycles 1-9 for the precipitate fraction of one sample of 0.01 M sodium citrate extraction.


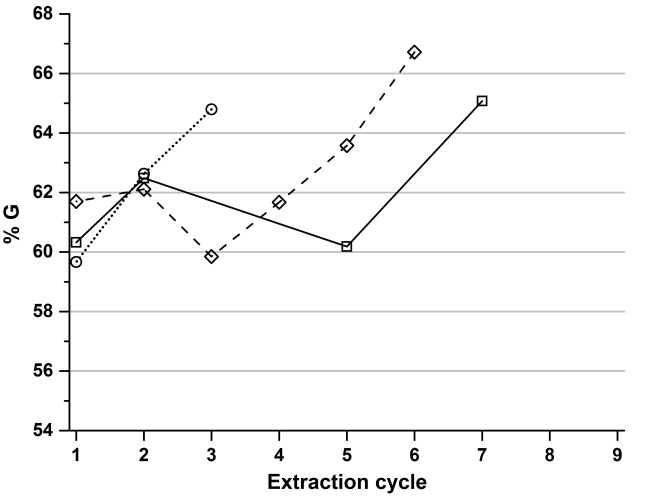


Online resource 2. Guluronic acid (G) content of the precipitated fraction determined by anion exchange chromatography for extractions at sodium citrate concentrations (
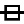
) 0.01 M, (
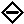
) 0.02 M and (
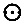
) 0.05 M.

| **Extraction cycle** | | **1** | **2** | **3** | **4** | **5** | **6** | **7** | **8** | **9** |
| --- | --- | --- | --- | --- | --- | --- | --- | --- | --- | --- |
| **% G** | **0.01 M** | 60.3  58.1  56.6 ±2.9 | 62.5  56.9  54.7 | -  60.2  58.0 | -  60.6  - | 60.2  62.9 ±0.7  - | -  64.8 ±0.4  - | 65.1  -  - | -  66.4 ±0.3  - | -  66.0  - |
|  | **0.02 M** | 61.7  55.4 ±1.0  54.1 ±0.8 | 62.1  56.9 ±0.2  55.4 ±0.4 | 59.8  61.3 ±0.4  - | 61.7  64.0 ±0.2  - | 63.6  64.2 ±0.4  - | 66.7  63.9 ±0.4  - | -  63.8 ±1.3  - | -  -  - | -  -  - |
|  | **0.05 M** | 59.7  59.7±0.6  59.2 ±0.0 | 62.6  62.4±0.3  60.8 ±0.2 | 64.8  63.0±0.2  61.2 ±0.1 | -  62.6±0.7  - | -  -  - | -  -  - | -  -  - | -  -  - | -  -  - |

**Online Resource 3.** Average G content of the precipitated fractions determined by anion exchange chromatography (top) with standard hydrolysis protocol using polyvalent cation removal, (middle) with mild hydrolysis protocol using polyvalent cation removal and (bottom) with mild hydrolysis protocol without polyvalent cation removal. Sodium citrate concentrations 0.01 M, 0.02 M and 0.05 M are listed. ± indicates standard deviation when applicable.


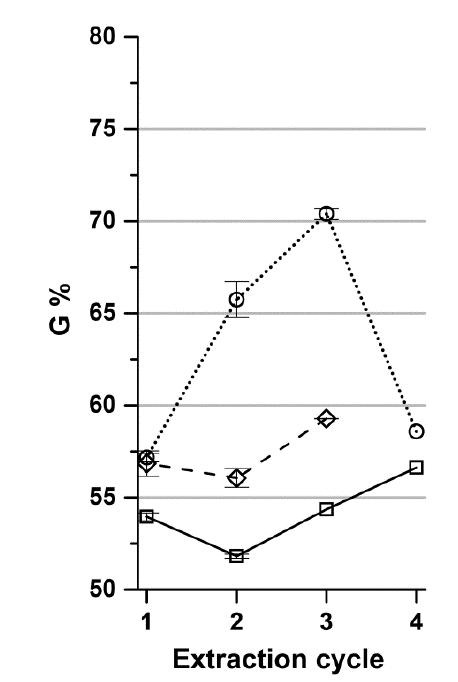


**Online Resource 4.** The content of G in the precipitated alginate from in each extraction cycle for extractions without polyvalent cation removal at sodium citrate concentrations (
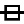
) 0.01 M, (
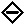
) 0.02 M and (
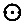
) 0.05 M. Error bars represent standard deviation of two independent replicates.

**
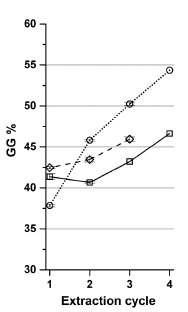
**

**Online Resource 5.** The content of GG dyads of the precipitated alginate in each extraction cycle for extractions without polyvalent cation removal at sodium citrate concentrations (
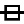
) 0.01 M, (
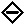
) 0.02 M and (
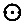
) 0.05 M. Error bars represents standard deviation of two independent replicates.


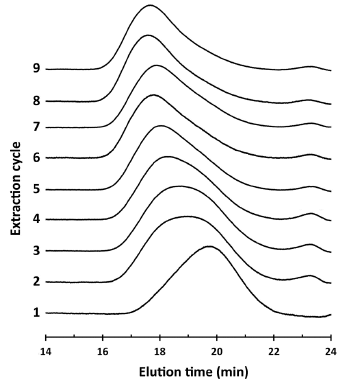


**Online Resource 6.** The SEC traces of the nine fractions of one sample from the extraction with 0.01 M sodium citrate.
